# Machine Learning‐Driven Discovery of Structurally Related Natural Products as Activators of the Cardiac Calcium Pump SERCA2a

**DOI:** 10.1002/cmdc.202400913

**Published:** 2025-02-06

**Authors:** Carlos Cruz‐Cortés, Eli Fernández‐de Gortari, Rodrigo Aguayo‐Ortiz, Jaroslava Šeflová, Adam Ard, Martin Clasby, Justus Anumonwo, L. Michel Espinoza‐Fonseca

**Affiliations:** ^1^ Center for Arrhythmia Research Department of Internal Medicine Division of Cardiovascular Medicine University of Michigan Ann Arbor MI-48109 USA; ^2^ International Iberian Nanotechnology Laboratory Braga 4715-330 Portugal; ^3^ Euskal Oxcitas Biotek SL Calle Lutxana 11 – DCHA 48008 Bilbao Spain; ^4^ Departamento de Farmacia Facultad de Química Universidad Nacional Autónoma de México Mexico City 04510 Mexico; ^5^ Department of Cell and Molecular Physiology Loyola University Chicago Maywood IL-60153 USA; ^6^ College of Pharmacy University of Michigan Ann Arbor MI-48109 USA; ^7^ Vahlteich Medicinal Chemistry Core University of Michigan Ann Arbor MI-48109 USA

**Keywords:** cardiac calcium pump, SERCA2a, small-molecule activator, machine learning, heart failure, small-molecule screening

## Abstract

A key molecular dysfunction in heart failure is the reduced activity of the cardiac sarcoplasmic reticulum Ca^2+^‐ATPase (SERCA2a) in cardiac muscle cells. Reactivating SERCA2a improves cardiac function in heart failure models, making it a validated target and an attractive therapeutic approach for heart failure therapy. However, finding small‐molecule SERCA2a activators is challenging. In this study, we used a machine learning‐based virtual screening to identify SERCA2a activators among 57,423 natural products. The machine learning model identified ten structurally related natural products from *Zingiber officinale*, *Aframomum melegueta*, *Alpinia officinarum, Alpinia oxyphylla*, and *Capsicum* (chili peppers) as SERCA2a activators. Initial ATPase assays showed seven of these activate SERCA at low micromolar concentrations. Notably, two natural products, Yakuchinone A and Alpinoid D displayed robust concentration‐dependent responses in primary ATPase activity assays, efficient lipid bilayer binding and permeation in atomistic simulations, and enhanced intracellular Ca^2+^ transport in adult mouse cardiac cells. While these natural products exert off‐target effects on Ca^2+^ signaling, these compounds offer promising avenues for the design and optimization of lead compounds. In conclusion, this study increases the array of calcium pump effectors and provides new scaffolds for the development of novel SERCA2a activators as new therapies for heart failure.

## Graphical abstract

Heart failure involves reduced SERCA2a activity, crucial for calcium regulation in heart cells. This study reports machine learning screening of 57,423 natural products, identifying several structurally related SERCA2a activators. Three compounds enhance calcium transport in cardiac cells, despite off‐target effects, offering promising hits for developing SERCA2a‐based heart failure therapies.

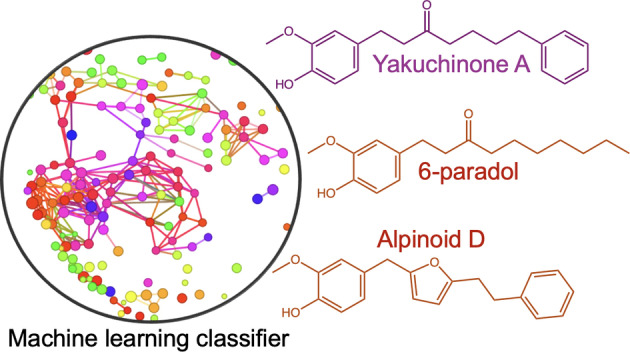

## Introduction

Machine learning is generating major inroads in drug discovery, and diverse studies are showing the success of this approach in enabling computer‐to‐benchside investigation of new therapeutic candidates.^[1]^ Traditionally, a major component for a successful drug discovery enabled by machine learning is the quality and quantity of molecular and structural information available to develop predictive structure‐activity relationships required for screening and de novo design. While machine learning has proven effective when applied to well‐characterized drug target spaces (i. e., the collection of targets for drugs),[^2^, ^3^, ^4^] its applicability to drug target spaces that are difficult to characterize has not been explored because of issues such as over‐fitting and futile learning layers.[^3^, ^5^] To overcome this limitation, we recently developed and validated a novel approach that combines machine learning and data augmentation for ligand‐based virtual screening of thousands of compounds.^[5]^ This validated model has allowed us to efficiently screen for active molecules using large chemical libraries.

The cardiac sarcoplasmic reticulum (SR) Ca^2+^‐ATPase (SERCA2a) is a prime example of a pharmacological target for which a drug space has proven challenging to characterize. SERCA2a is an ATP‐fueled pump that plays an essential role in normal cardiac function, actively transporting cytosolic Ca^2+^ into the SR during the diastolic phase of the cardiac cycle, relaxing muscle cells, and allowing the ventricles to fill with blood.^[6]^ The pathological dysregulation of this pump is a hallmark of heart failure (HF), the leading cause of morbidity and mortality in the United States, accounting for 1 in 4 deaths and costing the country about $219 billion each year in health care services, medicines, and loss in productivity.[^7^, ^8^] A key molecular dysfunction in HF involves insufficient SERCA2a expression and decreased PLN phosphorylation, leading to decreased SERCA‐mediated Ca^2+^ transport in cardiac cells.^[9]^ Reactivation of Ca^2+^ transport results in improved cardiac function in HF models.[^10^, ^11^, ^12^] Hence, SERCA2a is a well‐validated target, and its activation is an attractive goal for HF therapy.[^13^, ^14^, ^15^]

Biosensor‐based high‐throughput screening and medicinal chemistry approaches have been used to discover small‐molecule SERCA2a modulators.[^16^, ^17^, ^18^, ^19^, ^20^, ^21^] However, these studies have yielded only a handful of SERCA2a activators validated only in primary assays,[^16^, ^17^, ^18^, ^20^] or have failed to reproducibly activate SERCA2a activity in primary assays (e. g., Istaroxime).^[22]^ These limit the systematic interrogation of therapeutic modulation of cardiac calcium transport and their ability to engage SERCA2a in cardiac cells. Therefore, in this study, we used a machine learning‐based virtual screening approach we developed recently to identify small‐molecule SERCA2a activators from a dataset of 57,423 natural products. We chose to screen natural products because historically they have played a key role in drug discovery in several therapeutic areas,^[23]^ including cardiovascular disease (e. g., statins and digitalis).^[24]^ The model identified ten phenolic natural products from *Zingiber officinale*, *Aframomum melegueta*, *Alpinia officinarum, Alpinia oxyphylla*, and chili peppers from the genus *Capsicum* as SERCA2a activators. The activity of the predicted natural products was tested *in vitro* by using ATPase assays. These assays revealed that seven natural products activate SERCA2a potently in the low micromolar concentration range. Among these natural products, we discovered two natural products, Yakuchinone A and Alpinoid D, which bind and permeate across the lipid bilayer and are also potent effectors of SERCA2a‐mediated Ca^2+^ transport in cardiac myocytes. We discuss the potential off‐target effects of these natural products and future opportunities for the development of potent SERCA2a activators around these natural products.

## Results and Discussion

There are currently only a few validated SERCA2a activators reported in the literature.[^16^, ^17^, ^18^, ^22^] Therefore, the lack of sufficient training data for training the machine learning classifier is a challenge. Indeed, we have recently shown that model training using a small dataset performs poorly, i. e., the classifier has a near‐perfect performance when applied to the training set, but the performance gap is large when applied to a test set.^[5]^ The result is overfitting of the model, in which training with a limited dataset cannot be generalized and fits too closely to the training dataset.^[5]^ We recently demonstrated that ligand data augmentation helps overcome this issue and substantially improves the performance of the classifier with larger augmented training sets, exemplified by a narrower gap in the performance between the training and test sets. We performed data augmentation using a generative approach capable of breeding new structures starting from a seed compound by growing, mutating, or linking a predefined set of fragments that comply with the seed molecule.^[5]^ By using this approach, we maintain a low rate of activity cliffs while allowing the generation of a continuous SAR of SERCA2a activation in the augmented dataset that can be used to train the classifier model.

**Figure 1:**
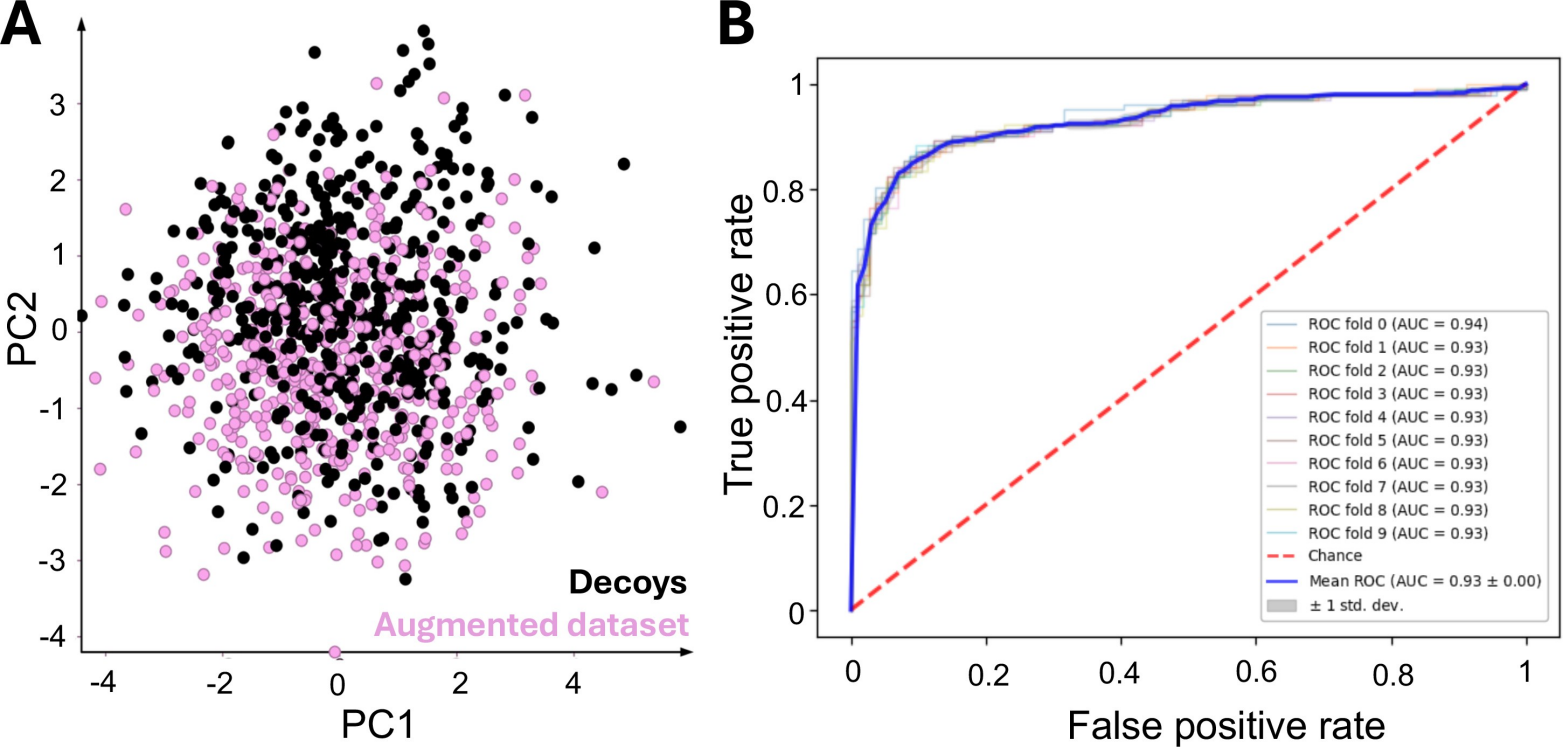
**Performance of the machine learning model trained with the augmented dataset of SERCA2a activators**. (A) A two‐dimensional molecular representation of the properties space by principal component analysis augmented dataset of SERCA2a activation is shown as pink circles, and the augmented dataset of decoys is shown as black circles. (B) We evaluated the performance of the machine learning model by using the ROC curve of the model trained with the augmented dataset of SERCA2a activators. In all cases, a value of one for both true and predicted labels is assigned to SERCA2a activators, whereas a value of zero is assigned to inactive molecules.

Molecular generators are prone to producing uncommon substructures, intricate chemical patterns, duplicate molecules, small diversity sets, or incorrect compounds. Therefore, we generated between 200 and 300 molecules using two hundred optimization steps over a hundred runs on average to ensure that the augmented dataset contains sufficient, yet accurate, structural information necessary to recapitulate the chemotype for activation of SERCA2a. Data curation produced a total of 575 molecules that represent the augmented dataset of SERCA2a activators to train the model. To complement the training set for supervised learning, we used 50 decoys per generated structure. We then randomly selected one of the 50 decoys obtained per generated structure, resulting in a final 1 : 1 rate of ‘active’ and ‘inactive’ (decoy) molecules, with a total of 1,150 molecules for both classes. This approach facilitates the identification of essential chemical features and increases the molecular complexity of the chemical space for SERCA2a activation. The result is a continuous map representing the virtual space occupied by SERCA2a activators and inactive molecules (decoys) (Figure 1A).

We trained the classifier model using the augmented dataset with the XGBoost method. We used this model because it was developed to control over‐fitting, improve performance, and increase the computing speed compared with other traditional non‐ensemble algorithms. We used a grid search with ten‐fold cross‐validation to tune the hyperparameters and validate the model. Analysis of the receiver‐operating characteristic (ROC) curve showed that the resulting classifier model trained with the augmented dataset improves the performance of the classifier, illustrated by a high mean accuracy (~91 %), a low mean standard deviation of the model (2.7 %) and a high ratio of true positives to false positives (ratio of 9 : 1) (Figure 1B and Table 1). These findings also agree with our previous studies showing that the use of data augmentation produces a robust machine‐learning classifier.^[5]^ These results show that virtual augmentation of the chemical space recapitulates the fundamental chemical properties that are required for SERCA2a activation.

**Table 1:** Summary of performance of the machine learning classifier trained with the augmented dataset of SERCA2a activators.

True Negatives	101
False Negatives	13
False Positives	11
True Positives	91
Mean Accuracy	91.5 %
Mean SD	2.7 %
Best Accuracy	92.2 %

**Figure 2:**
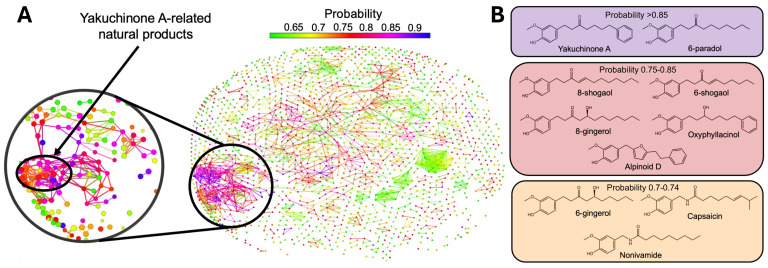
**A t‐SNE dimensionality reduction and network representation of the natural products**. (A) We used t‐SNE as a technique for nonlinear dimensionality reduction used for embedding the high‐dimensional chemical space of SERCA2a activation in a low‐dimensional space. The space represents only natural products with a predicted probability as SERCA2a activators larger than 0.6. Natural products are represented as nodes linked by their structural similarity. Each node is color‐coded by the output probability of SERCA2a activation calculated by the classifier. The model predicted a cluster of natural compounds that are structurally related to Yakuchinone A. (B) After applying toxicity and drug‐likeness filters, we identified ten natural products as SERCA2a activators. We show the structures and predicted probabilities of these natural compounds predicted by the machine learning model.

We next used the trained machine learning model to screen a dataset of 57,423 natural products^[25]^ and classified them by using dimensionality reduction and visualization with the T‐distributed stochastic neighbor embedding (t‐SNE).^[26]^ Based on our recent work,^[5]^ we selected drugs with class probability values between 0.7 and 0.9 and neighbor counts (e. g., chemically similar molecules) larger than three as initial filtering parameters to identify compounds that activate SERCA2a. After applying filters to select drug‐like molecules (see experimental section), our model predicted several dense clusters of molecules with consistent probability values >0.7, which we interpret as a conservation of the pharmacophoric elements representing SERCA2a activation (Figure 2A). This cluster contains a family of phenolic natural products chemically related to Yakuchinone A (p>0.85; Figure 2B), a natural product isolated from the fruit of *Alpinia oxyphylla*. The model predicted nine natural products structurally related to Yakuchinone A: 6‐paradol, 6‐shogaol, 8‐shogaol, 6‐gingerol, 8‐gingerol, oxyphyllacinol, Alpinoid D, nonivamide, and capsaicin (Figure 2B). Some of these natural products have been reported to have several pharmacological effects, including antioxidant,[^27^, ^28^, ^29^] anti‐hyperglycemic,^[30]^ and anti‐inflammatory[^29^, ^31^] properties. The findings are significant because natural phenols are known to modulate SERCA activity,^[32]^ and because two natural products predicted by the machine learning classifier, 6‐gingerol, and capsaicin, have been proposed as activators of the skeletal muscle and cardiac calcium pump isoforms.[^33^, ^34^, ^35^] Therefore, we tested *in vitro* the effects of these ten natural products on the ATPase activity of the cardiac SERCA2a.

Compound screening assays for hit discovery are typically run at compound concentrations between 1 and 10 μM.^[36]^ Since our goal is to identify molecules that are active in this concentration range, we first tested the activity of the compounds at 1 and 10 μM at seven Ca^2+^ concentrations (see methods). Our initial screening showed that all ten natural products have marginal (<5 %) or no effect on the ATPase activity of SERCA2a at a compound concentration of 1 μM (Table 2). Nevertheless, at a compound concentration of 10 μM a clear activating effect of up to 31 % was observed, only three natural products, 6‐gingerol, nonivamide, and capsaicin did not stimulate SERCA2a ATPase activity at a compound concentration of 10 μM (Table 2). The initial screening indicates that the model correctly predicted the activity of seven molecules, and the predictive power is below that of the trained model, i. e., 70 % success rate based on experimental validation *vs* ~90 % performance of the trained model (Figure 1B). We note, however, that the probability of 6‐gingerol, nonivamide and capsaicin as SERCA2a activators is lower than other compounds (Figure 2B), which is still consistent with the initial experimental validation. Previous studies have shown that 6‐gingerol activates the ATPase activities of the calcium pump in the low micromolar concentration range,[^33^, ^34^] but we could not reproduce these findings. Likewise, studies using SR membranes showed that capsaicin activates SERCA2a at concentrations near the 10 μM concentration range,^[35]^ but we found no measurable changes in SERCA2a activity at this compound concentration (Table 2). It is possible that 6‐gingerol and capsaicin do not activate SERCA2a in our assays probably because of species‐specific differences, e. g., pig (this study) *vs* dog/rabbit tissue.[^33^, ^35^] However, we rule out this possibility because independent studies have shown that 6‐gingerol does not have a significant effect on rabbit SERCA1a activity at low concentrations of the compound (i. e., ≤10 μM),[^37^, ^38^] and because calcium pump effectors are generally not species‐ or isoform‐specific.[^5^, ^21^, ^22^]

**Table 2:** Effects of the natural products predicted by the model on the ATPase activity of SERCA2a.

Natural product	SERCA2a activity at 1 μM (%)	SERCA2a activity at 10 μM (%)
Yakuchinone A	97.4 ±2.6	119.6±4.4
6‐paradol	103.9±4.5	131.3±6.2
6‐shogaol	95.7±1.2	106.37±3.2
8‐shogaol	103.8±5.0	117.8±4.6
6‐gingerol	99.1±3.5	103.6±2.0
8‐gingerol	100.9±1.7	117.56±1.6
Oxyphyllacinol	99.8±1.8	120.4±3.6
Alpinoid D	101.2±4.6	126.5±2.7
Capsaicin	96.3±2.7	97.4±1.9
Nonivamide	96.7±5.3	94.2±2.6

Data are reported as average±SEM (N=3). Reported values were obtained relative to the untreated microsomes.

**Figure 3:**
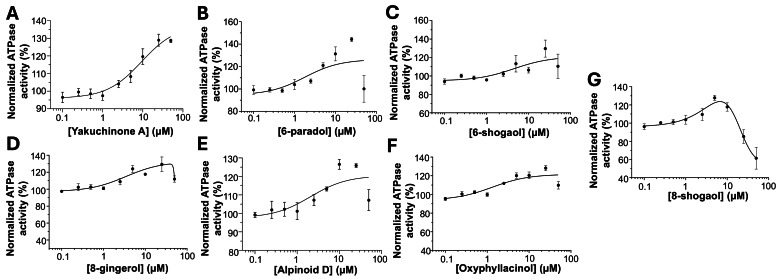
**Effects of seven natural products on the ATPase activity of SERCA2a**. We show the nine‐point concentration‐response curves of the natural products (A) Yakuchinone A, (B) 6‐paradol (C) 6‐shogaol, (D) 8‐gingerol; (E) Alpinoid D, (F) Oxyphyllacinol, and (G) 8‐shogaol against cardiac SERCA2a. In all cases, the activity of the compounds at each concentration was obtained from the Ca^2+^‐concentration‐dependent SERCA2a activity and normalized relative to the untreated control. Data are reported as average ± SEM (N=3).

We next obtained the concentration‐response curves at nine concentrations (0.1–50 μM) of the seven natural products that stimulated SERCA2a at a concentration of 10 μM in our initial primary assays. The natural products tested are Yakuchinone A, 6‐paradol, 6‐shogaol, 8‐shogaol, 8‐gingerol, oxyphyllacinol, and Alpinoid D. The ATPase activity is represented as the % of the change in maximal activity (V_max_) relative to the activity of untreated SERCA2a (negative control). When describing the data, we make a distinction between ‘observed’ data (i. e., data points) and ‘estimated’ data (i. e., values derived from the fitted model data). Yakuchinone A showed a substantial effect on the activity of SERCA2a, with an observed stimulation of SERCA2a activity of ~27 % at compound concentrations >25 μM, and an estimated EC_50_=9.3 μM (Figure 3A). 6‐paradol, another compound predicted with high probability as a SERCA2a activator, is a potent SERCA2a activator at compound concentrations between 5 μM (increase in V_max_ ~20 %) and 25 μM (increase in V_max_ ~43 %) (Figure 3B). However, the stimulatory activity of 6‐paradol is lost at a compound concentration of 50 μM (Figure 3B). The natural products 6‐shogaol and 8‐gingerol displayed a similar stimulatory activity on SERCA2a, with an observed maximal effect on the V_max_ at a compound concentration of 25 μM (increase in V_max_ ~30 %) and a drop in stimulatory activity at a concentration of 50 μM (Figure 3C and D). A similar behavior was observed for the natural products Alpinoid D and oxyphyllacinol, with an observed maximal effect on the V_max_ (increase by 25 %) at a compound concentration of 10 μM and 25 μM, respectively (Figure 3E and F). A drop in ATPase activity was also observed for these natural products at a compound concentration of 50 μM (Figure 3E and F). 8‐shogaol induced paradoxical effects on SERCA2a ATPase, activating the pump at compound concentrations ≤10 μM (e. g., increase V_max_ by 25 % at 5 μM) and inhibiting SERCA2a at compound concentrations >25 μM (e. g., decrease in V_max_ by 40 % at 50 μM) (Figure 3G). We found that except for Yakuchinone A, the natural products tested here follow a bell‐shaped concentration‐response behavior. A possible explanation for this effect is self‐aggregation because these molecules are hydrophobic and share structural similarities with molecules that are known to aggregate at concentrations >10 μM.^[39]^ We have also recently shown that ATP is both a substrate and a modulator of SERCA2a. Therefore, it is also possible that at high concentrations, the natural products could impair the ATP‐mediated activation of SERCA2a and prevent the formation of catalytically favorable conformations.^[22]^ It is also possible that at high concentrations, these natural products can intercalate into lipid bilayers, disrupting the lipid microenvironment around SERCA2a, and inhibiting the pump's activity.^[40]^

Targeting the cardiac SERCA2a pump requires molecules that permeate through and be retained at the membrane to reach the SR.^[41]^ Therefore, we first used single‐molecule atomistic simulations to evaluate whether these molecules can bind and permeate through a lipid bilayer; see supplementary materials for details on the experimental procedures. The ATPase activity assays showed that only Yakuchinone A, 6‐paradol and Alpinoid D display a robust concentration‐response relationship at compound concentrations ≤25 μM (Figure 3A, B and D). Therefore, we selected these molecules for further evaluation using *in silico* studies. Unrestrained single‐molecule molecular dynamics (MD) simulations of the activators embedded in a lipid bilayer show that the molecules remain bound to the membrane and localize at the interface between the polar and nonpolar regions of the membrane, i. e., between 4–8 Å below the lipid headgroups (Figures S1–S3, Supplementary information). Lipid‐bilayer crossing free‐energy profiles showed that all three molecules are hydrophobic and interact favorably with the lipid bilayer, with free energies of binding (ΔG_bind_) between −3.5 and −6.5 kcal mol^−1^ (Figures S1–S3, Supplementary information).

**Figure 4:**
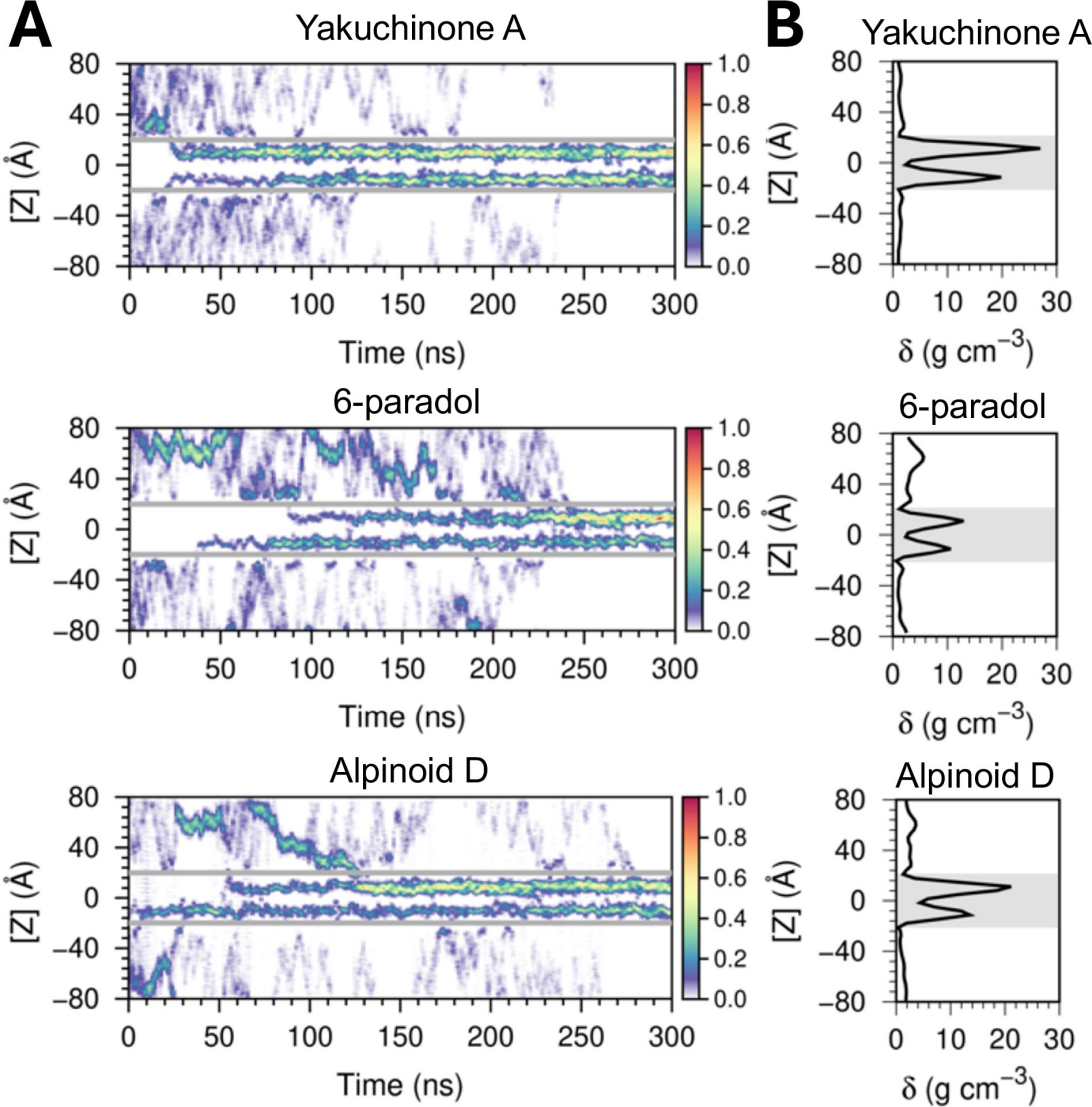
**Ensemble‐based atomistic simulations of ligand–membrane interactions of the SERCA2a activators Yakuchinone A, 6‐paradol, and Alpinoid D**. (A) Time‐dependent behavior of the natural compounds in a lipid–water environment. The simulations capture the spontaneous insertion of small molecules into the membrane as well as ligand–ligand interactions during the 300 ns of simulation time. The scale represents the occupancy fraction of the natural products in the lipid‐water system. The gray horizontal lines show the boundaries of the lipid bilayer. (B) The relative density (δ) profiles of SERCA2a activators in a lipid–water system along the z‐axis of the system. The gray shaded area shows the boundaries of the lipid bilayer.

We performed ensemble‐based atomistic simulations^[42]^ of Yakuchinone A, 6‐paradol, and Alpinoid D to complement single‐molecule MD simulations and better capture the behavior of these natural products in a lipid‐water environment and in real‐time. We used these simulations because previous studies by our group have shown that ensemble‐based atomistic simulations of ligand‐membrane provide unique insights into ligand‐membrane interactions not detected in single‐molecule profiling.^[41]^ The ensemble‐based simulations capture the spontaneous insertion of Yakuchinone A, 6‐paradol, and Alpinoid D into the lipid bilayer (Figure 4A–C), in agreement with the single‐molecule simulations. However, the ensemble simulations revealed differences in the ligand‐ligand and ligand‐membrane interaction profiles among these SERCA2a activators. The time‐dependent analysis of Yakuchinone A in a lipid‐water environment showed that most of the ligand molecules bind rapidly to both leaflets of the lipid bilayer (e. g., at *t*=20 ns), with a small number of molecules remaining in the aqueous phase at t>20 ns (Figure 4A, top panel). Analysis of the relative density calculated from the 300‐ns trajectory clearly illustrates this behavior (Figure 4B, top panel), showing a high density of molecules embedded in the lipid bilayer (≥20 g cm^−3^) and a low density of molecules in the aqueous phase (<2 g cm^−3^). Conversely, we found that 6‐paradol diffuses slower into the membrane at the timescale used here. Specifically, 6‐paradol binds to one of the leaflets of the lipid bilayer at t=40 ns, and both leaflets of the membrane at t=90 ns (Figure 4A, middle panel). The relative density profile of 6‐paradol showed that the relative density of molecules embedded in the lipid bilayer is much lower (≤12 g cm^−3^) than that observed for Yakuchinone A (Figure 4B, middle panel). Conversely, the relative density of 6‐paradol molecules in the aqueous phase is higher, with a maximal relative density of ~5 g cm^−3^ (Figure 4B, middle panel). Finally, Alpinoid D binds rapidly to the lipid bilayer and behaves similarly to Yakuchinone A, although with less density of molecules bound to the lipid bilayer and slightly more ligand density remaining in the aqueous phase (Figure 4A and B, lower panels). Overall, these findings indicate that while the ligand‐lipid interaction profiles are different among these activators, the three natural products preferably bind to the lipid bilayer. This is important because recent docking studies have suggested that activators, including natural phenolic compounds, bind to the cytosolic domain of SERCA2a.[^20^, ^37^] However, our simulations indicate that these compounds preferably interact with the lipid bilayer, thus suggesting that the effector site is in the transmembrane domain of the pump. Future studies are required to test this hypothesis and clarify the location of the binding site for these natural products.

**Figure 5:**
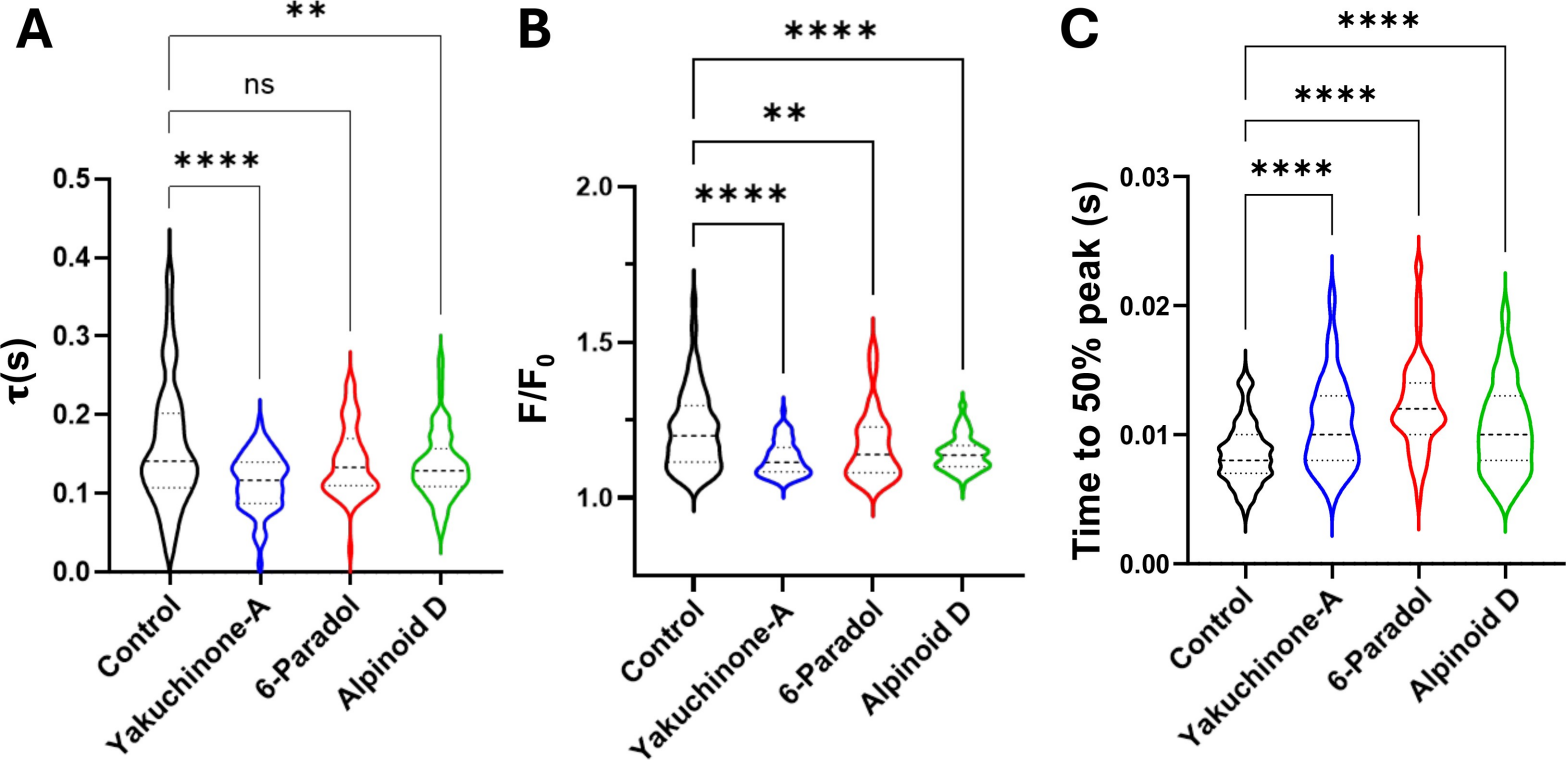
**Effects of SERCA2a activators on the intracellular calcium transient in adult mouse cardiac cells**. We measured the effects of Yakuchinone A, 6‐paradol, and Alpinoid D on (A) Ca^2+^ transient decay (τ) and (B) Ca^2+^ transient amplitude and (C) time to 50 % peak measured in cardiac myocytes. Ca^2+^ transients were recorded at 1‐Hz field stimulation at a compound concentration of 10 μM. For comparison, we performed experiments using untreated myocytes as a control. Data are presented as a violin plot, where dashed lines represent quartiles, full lines represent the median, and widths represent the number of individuals with the same value of the measured parameter (N=8 mice). Statistical differences were tested using ANOVA followed by Dunnett's post‐hoc test; **p<0.01, **** *p*<0.0001.

The ATPase assays show that Yakuchinone A, 6‐paradol, and Alpinoid D stimulate SERCA2a activity, but the simulations show that these molecules interact differently with the lipid bilayer. Previous studies by our group have shown that ligand‐lipid interaction profiles correlate with target engagement in cardiomyocytes.^[41]^ Therefore, we expect that Yakuchinone A will activate SERCA2a more efficiently than Alpinoid D and 6‐paradol in live cells. To test this hypothesis, we used single‐cell Ca^2+^ imaging to establish the effects of the three natural products on intracellular Ca^2+^ dynamics in cardiac myocytes isolated from mice. Ca^2+^ transients were analyzed at 1 Hz field stimulation. We first analyzed the Ca^2+^ transient decay as a proxy for SERCA2a engagement because previous studies by our group have shown that small‐molecule activators shorten the duration of the Ca^2+^ transient in cardiac cells.^[41]^ We found that the Ca^2+^ transient decay, τ, is significantly faster in myocytes treated with Yakuchinone A (*p*<0.0001 vs untreated control) and Alpinoid D (*p*<0.01 *vs* untreated control), but 6‐paradol has no effect on τ at a compound concentration of 10 μM (Figure 5A). While there are no crystal structures of SERCA2a bound to activators reported in the literature, the existing crystallographic evidence suggests that most effector sites are located in the transmembrane domain of the pump.^[43]^ This suggests that the ability of natural products to partition into the lipid bilayer surrounding SERCA2a could significantly impact their efficacy in cardiac myocytes. The differences in activity can be explained in part by the ligand‐lipid interaction profiles of the natural products (Figure 4), where 6‐paradol interacts less efficiently with the lipid bilayer compared to Yakuchinone A and Alpinoid D.

We have shown that small‐molecule SERCA2a activation shortens the duration of the Ca^2+^ transient in human iPSC‐derived cardiomyocytes,^[41]^ which is consistent with the effects of Yakuchinone A and Alpinoid D observed in this study (Figure 5A). In mouse cardiac myocytes, the Ca^2+^ transient decay reflects the combined activity of SERCA2a and the Na^+^‐Ca^2+^ exchanger (NCX1), the former contributes to ~90 % of Ca^2+^ uptake in mice.^[44]^ Therefore, we can infer that the effect on the Ca^2+^ transient decay induced by Yakuchinone A and Alpinoid D is due to SERCA2a engagement in cardiac myocytes. We have also previously shown that small‐molecule SERCA2a activation increases the Ca^2+^ transient amplitude in cardiomyocytes, but we observed the opposite effect by all three natural products tested here. Compared to the untreated control, the stimulated Ca^2+^ transient amplitude in myocytes from mice was significantly decreased by all three natural products, with Yakuchinone A and Alpinoid D exerting the most significant effect (*p*<0.0001 *vs* untreated control; Figure 5B). A possible explanation for the effects of these compounds on the Ca^2+^ transient amplitude is the inhibition of the L‐type Ca^2+^ channel. Indeed, previous studies have shown that the closely related compound 6‐gingerol significantly reduces Ca^2+^ transients of cardiomyocytes primarily by inhibition of the L‐type Ca^2+^ channel.^[45]^ Given their high similarity with 6‐gingerol, it is very likely that Yakuchinone A, 6‐paradol, and Alpinoid D also inhibit the L‐type Ca^2+^ channel in cardiac myocytes. These findings are important because this channel is the primary route for Ca^2+^ entry into myocytes, and plays a key role in initiating the excitation–contraction (E−C) coupling in cardiac cells.^[46]^ Analysis of the Ca^2+^ transient time to 50 % peak showed a significant slowing of calcium transient development upon treatment of cardiac myocytes with Yakuchinone A, 6‐paradol, and Alpinoid D (Figure 5C). These observations suggest that these compounds have off‐target effects that affect the initiation of the E−C coupling in cardiac myocytes, and these effects must be addressed in future hit‐to‐lead optimization campaigns.

In summary, we used a combination of data augmentation and machine learning to screen for SERCA2a activators in a dataset of 57,423 natural products. The model identified ten natural products from *Zingiber officinale*, *Aframomum melegueta*, *Alpinia officinarum, Alpinia oxyphylla*, and chili peppers from the genus *Capsicum* as SERCA2a activators. Primary ATPase assays showed that 7 out of the 10 natural products identified by the machine learning classifier stimulate SERCA2a activity in a concentration‐response manner. The ATPase assays also showed that the natural product, 8‐shogaol, is both an activator and inhibitor of SERCA2a at different concentrations of the compound. We selected three natural products based on their ATPase activity profiles for further *in silico* permeability studies and cell‐based secondary screening assays. These studies showed that two natural products, Yakuchinone A and Alpinoid D, bind and permeate across the lipid bilayer efficiently, and significantly stimulate SERCA2a‐mediated Ca^2+^ transport in cardiac myocytes isolated from mice. While both natural products are potent SERCA2a activators, the cell‐based assays revealed off‐target effects in cardiac myocytes, including potential inhibition of the L‐type Ca^2+^ channel. These off‐target effects need to be studied in detail in the future, e. g., by using single‐cell electrophysiology assays, and efforts to eliminate these liabilities must be addressed in future hit‐to‐lead development of SERCA2a activators. In conclusion, this study increases both the array of calcium pump effectors and provides new simple scaffolds for the development of potent SERCA2a activators as new therapies for heart failure.

## Experimental Section

*Data augmentation and machine model training*. We have recently published the machine‐learning virtual screening method used here,^[5]^ but we now retrained it to screen for SERCA2a activators. For the initial training of the model, we used validated activators that have been reported in the literature.[^16^, ^17^, ^18^, ^22^] All compounds’ SMILES (simplified molecular‐input line‐entry system) were verified and transformed to canonical SMILES using DataWarrior.^[47]^ To further increase the molecular diversity of putative effectors, we generated decoys using the DeepCoy generator.^[48]^ Canonical SMILES were input to the Chem library from RDKit to calculate chemical MACCS (Molecular access system) keys^[49]^ and the extended connectivity radius of two fingerprints. All representations were concatenated with seven molecular and physicochemical properties and standardized using the Z‐score normalization using StandardScaler in Scikit‐learn as used earlier by us.^[50]^ We generated an augmented training dataset of SERCA2a activators using the CReM package^[51]^ by using the grow function with a conservative radius of three and the CHEMBL fragment dataset. The resulting dataset contained 1,150 molecules for activators and inactive molecules, which were included in the training set labeled as the source molecule. The initial and augmented datasets of SERCA2a activators were subjected to stratified splitting as 85 % for the training set and 25 % for the testing set with a constant random state. The augmented dataset was used to train the XGBoost method. The model was fed with the preprocessed label data for a grid search of hyperparameters and 10‐cross‐validation using recall as a scoring function. The performance of the model was evaluated using the ROC and accuracy.^[50]^

*Screening and hit selection*. The model trained with the augmented training dataset was used here to probe the map of SERCA2a activation using 57,423 molecules from the traditional Chinese medicine Database@Taiwan.^[25]^ We performed a dimensionality reduction and visualization for the FDA‐approved molecules using T‐distributed stochastic neighbor embedding (t‐SNE)^[26]^ on molecules PathFp descriptors in DataWarrior.^[47]^ The result is a similarity map, where each node represents a natural product and each edge represents the Tanimoto similarity^[52]^ between two adjacent nodes. By using this approach, a pair of compounds directly connected and with a significant difference in probability is considered as an activity cliff, whereas a small difference in predicted probability indicates a continuous SAR, thus indicating a space occupied by SERCA2a activators. We selected molecules using a model classification probability larger than 0.7 and neighbor count equal to or larger than 3 from a similarity network as initial criteria for hit filtering.^[5]^ We applied a more stringent criterion for hit selection using pre‐trained filters to eliminate toxic compounds, including those having mutagenic, carcinogenic, and adverse effects on reproduction. Additional filters included selecting molecules with ≤10 rotatable bonds, a calculated LogP <5, and consensus drug‐likeness based on the criteria established by Lipinsky,^[53]^ Ghose,^[54]^ Veber,^[55]^ Egan,^[56]^ and Muegge.^[57]^

*Chemicals*. All natural products used in this study were purchased from MedChemExpress (NJ, USA) at ACS quality (purity>95 % by HPLC): Yakuchinone A, 1‐(4‐hydroxy‐3‐methoxyphenyl)‐7‐phenylheptan‐3‐one; 6‐paradol, (1‐(4‐hydroxy‐3‐methoxyphenyl)decan‐3‐one); 6‐shogaol, (*E*)‐1‐(4‐hydroxy‐3‐methoxyphenyl)dec‐4‐en‐3‐one; 8‐shogaol, (E)‐1‐(4‐hydroxy‐3‐methoxyphenyl)dodec‐4‐en‐3‐one; 6‐gingerol, (5S)‐5‐hydroxy‐1‐(4‐hydroxy‐3‐methoxyphenyl)decan‐3‐one; 8‐gingerol, (5S)‐5‐hydroxy‐1‐(4‐hydroxy‐3‐methoxyphenyl)dodecan‐3‐one; oxyphyllacinol, 4‐(3‐hydroxy‐7‐phenylheptyl)‐2‐methoxyphenol; nonivamide, N‐[(4‐hydroxy‐3‐methoxyphenyl)methyl]nonanamide; capsaicin, (E)‐N‐[(4‐hydroxy‐3‐methoxyphenyl)methyl]‐8‐methylnon‐6‐enamide; Alpinoid D, 2‐methoxy‐4‐[[5‐(2‐phenylethyl)furan‐2‐yl]methyl]phenol.

*Isolation of enriched SERCA2a microsomes*. Pig hearts were obtained post‐euthanasia from the University of Michigan Unit for Laboratory Animal Medicine and placed in a cardioplegic solution (280 mM glucose, 13.44 mM KCl, 12.6 mM NaHCO_3_, and 34 mM mannitol). Left ventricles free walls were obtained, minced, and homogenized with a cold buffer that contained 9.1 mM NaHCO_3_, 0.9 mM Na_2_CO_3,_ and a cocktail of proteases inhibitors (Sigma, St. Louis, MO); the mixture was centrifuged at 6,500 *g* for 30 minutes at 4 °C to remove debris. The supernatant was filtered, collected, and centrifuged at 14,000 *g* for 30 min at 4 °C. The collected filtrate was centrifuged at 47,000 *g* for 60 min at 4 °C. The pellet was resuspended in a solution containing 0.6 M KCl and 20 mM Tris (pH=6.8). The suspension was centrifuged at 120,000 *g* for 60 min at 4 °C, and the pellet was resuspended in a solution containing 0.3 M sucrose, 5 mM MOPS, and protease inhibitors (pH=7.4). The protein concentration of the SR microsomal fraction was determined using the PierceTM Coomassie plus assay kit (Thermo‐Fisher Scientific, Waltham, MA). The microsomal membranes were aliquoted, frozen in liquid nitrogen, and stored at −80 °C.

*ATPase activity assays*. We performed SERCA2a activity assays using an enzyme‐coupled NADH‐linked ATPase activity assay described previously.^[5]^ Briefly, we measured the activity of Ca^2+^ ATPase in μmol min^−1^ mg^−1^ from the decrease in absorbance of NADH at 340 nm at 25 °C in a 96‐well format using a Synergy H1 (BioTek, Winooski, VT) microplate reader. Each well contained a 200 μl final volume of assay buffer containing SERCA2a buffer (50 mM MOPS, 100 mM KCl, 5 mM MgCl_2_, and 1 mM EGTA, pH=7), 5 U lactate dehydrogenase, 5 U pyruvate dehydrogenase, 1 mM phosphoenolpyruvate, 5 mM ATP, 0.2 mM NADH, 2 μg of microsomal suspension, 2 μM of Ca^2+^ ionophore A23187, and seven free Ca^2+^ concentrations. Each concentration of the compounds tested here was calculated to a final volume of 200 μl. We incubated the small molecules for 30 minutes at 25 °C with the reaction mixture. Concentration‐response curves for each compound were constructed with the data from [Ca^2+^]‐dependent SERCA2a activity curves performed at compound concentrations of 0.1–50 μM. The final free Ca^2+^ concentrations were calculated using MaxChelator,^[58]^ and were achieved by using twelve individual stock CaCl_2_ solutions. Each plate included untreated microsomes (negative control), as well as microsomes treated with thapsigargin (inhibition control) and CDN1163 (activation control).^[22]^ To account for biological variability, we use microsomal fractions obtained from three pig hearts. In all cases, the maximal activity (V_max_) values were normalized relative to the untreated microsomes.

*Ensemble‐based MD simulations of unbound natural products in a lipid‐water environment*. The simulations were performed as previously described by us.^[41]^ Briefly, a total of 10 molecules of each compound were randomly placed in the water phase of a system containing a POPC lipid bilayer consisting of 250 lipid molecules and about 34,300 water molecules. The system was energy minimized and equilibrated during 1 ns using NVT and NPT ensembles. The equilibrated system was subsequently submitted to a 300‐ns NPT production run using the previously described parameters. The density profiles were computed with the *density* built‐in tool of GROMACS 5.1.4, while the center of mass position of the molecule in the z‐axis of the membrane was calculated with MDAnalysis python library.^[59]^ Graphs were made using Gnuplot 5.0^[60]^; visualization was performed using PyMOL.^[61]^

*Single‐cell Ca* ^*2+*^ *imaging*. All animal studies were conducted with approval from the University of Michigan Institutional Animal Care and Use Committee protocol PRO00010664. Myocytes were isolated using a modified Langendorff technique. Ventricular myocytes were isolated and prepared for Ca^2+^ imaging as described in the Supplementary Information. The measurement of intracellular Ca^2+^ transients was performed using an Ionoptix recording system. Culture media was removed, and the myocyte‐plated coverslips were transferred into Tyrode solution containing the 140 mM NaCl, 4 mM KCl, 1 mM MgCl_2_, 10 mM HEPES, 10 mM Glucose, 1 mM CaCl_2_; the pH was adjusted to 7.4 with NaOH. Cells were loaded with 5 μM Fura2‐AM and 2.5 mM probenecid followed by 2 washouts of 10 min each for de‐esterification with Tyrode solution. Coverslips were placed in an Ionoptix rapid change stimulation chamber and perfused with Tyrode solution at 37 °C; Yakuchinone A, 6‐paradol, and Alpinoid D were tested at a concentration of 10 μM. A temperature of 37 °C was maintained using a mTC3 micro temperature controller. We used an inverted Fluorescence Microscope Nikon ECLIPSE Ti Series and a 40x/1.30 oil Nikon objective for data acquisition. Fura‐2 AM excitation wavelengths, 340 nm and 380 nm were generated using an IonOptix HyperSwitch. Cells were paced at 1 Hz with the IonOptix Myopacer. A minimum of 10 transients per myocyte were used for ensemble averaging and analyzed using the Ionwizard software (IonOptix LLC, Milton, MA).

*Statistical analysis*. All results are presented as mean ± standard error of the mean (SEM). Significance was evaluated using a two‐way analysis of variance (ANOVA) followed by Dunnett's post‐hoc test to analyze differences between the control and multiple treatments. We used 95 % confidence intervals around the differences between the groups for the post‐hoc test. Two‐sided *p* values were used, and α‐level <0.05 was considered significant.

## Conflict of Interests

The authors declare no competing financial interests.

1

## Supporting information

As a service to our authors and readers, this journal provides supporting information supplied by the authors. Such materials are peer reviewed and may be re‐organized for online delivery, but are not copy‐edited or typeset. Technical support issues arising from supporting information (other than missing files) should be addressed to the authors.

Supporting Information

## Data Availability

All data needed to evaluate the conclusions in the paper are present in the paper and/or the Supplementary Materials. In addition, data and code used in analyzing the dataset are available as described in the Methods section. Additional data related to this paper may be requested from the corresponding author.
