# Supplementary material for: Machine Learning‐Driven Discovery of Structurally Related Natural Products as Activators of the Cardiac Calcium Pump SERCA2a
Source: ChemMedChem. 2025 Feb 6;20(9):e202400913. doi: 10.1002/cmdc.202400913 (PMC12058239; doi:10.1002/cmdc.202400913)
Supplement: Supplementary file 1 — Supporting Information [file CMDC-20-e202400913-s001.pdf]

ChemMedChem

Supporting Information

Machine Learning-Driven Discovery of Structurally Related Natural Products as Activators of the Cardiac Calcium Pump SERCA2a

Carlos Cruz-Cortés, Eli Fernández-de Gortari, Rodrigo Aguayo-Ortiz, Jaroslava Šeflová, Adam Ard, Martin Clasby, Justus Anumonwo, and L. Michel Espinoza-Fonseca*

Supporting Information

Machine learning-driven discovery of structurally related natural products as activators of the cardiac calcium pump SERCA2a

Carlos Cruz-Cortes,[1] Eli Fernández-de Gortari,[2,3] Rodrigo Aguayo-Ortiz,[4] Jaroslava Šeflová,[5] Adam Ard,[6,7] Martin Clasby,[6,7] Justus Anumonwo,[1] and L. Michel Espinoza-Fonseca[1,*]

[1]Center for Arrhythmia Research, Department of Internal Medicine, Division of Cardiovascular Medicine, University of Michigan, Ann Arbor, MI 48109, USA
[2]International Iberian Nanotechnology Laboratory, Braga, 4715-330, Portugal
[3]Euskal Oxcitas Biotek SL, Calle Lutxana 11 - DCHA. 48008, Bilbao, Spain
[4]Departamento de Farmacia, Facultad de Química, Universidad Nacional Autónoma de México, Mexico City 04510, Mexico
[5]Department of Cell and Molecular Physiology, Loyola University Chicago, Maywood, IL 60153, USA
[6]College of Pharmacy, University of Michigan, Ann Arbor, MI 48109, USA
[7]Vahlteich Medicinal Chemistry Core, University of Michigan, Ann Arbor, MI 48109, USA

*Corresponding author: L. Michel Espinoza-Fonseca
Email: lmef@umich.edu

This PDF file includes:

Extended methods
Supplementary Figures S1 to S3
Supplementary references

Extended methods

<u>*Single-molecule bilayer-crossing profiles of SERCA2a activators*</u>**.** The 3-D structures of Yakuchinone A, 6-paradol, and Alpinoid D were retrieved from PubChem.[1] Geometry optimization was performed using the MMFF94s force field implemented in the *obminimize* module of OpenBabel.[2] We used the PerMM server[3] with the "Drag" method to calculate the pathway of translocation across a lipid bilayer of 1,2-dioleoyl-*sn*-glycero-3-phosphocholine (POPC) at 300 K and pH 7.1. We chose POPC as a lipid model because this lipid is predominantly found in the membrane of both the sarcolemma and the SR membrane in muscle cells.[4-5] Umbrella sampling simulations were carried out across a POPC membrane for each of the compounds with the AMBER99SB-ILDN force field[6] implemented in GROMACS 5.1.4.[7] Ligand topologies and parameters were generated with the ACPYPE interface[8] using AM1-BCC method to compute partial charges. POPC lipid parameters were taken from the Slipids (Stockholm Lipids) force field.[9] For the system setup, we set the molecules approximately 4.0 nm away from the center of mass of a pre-equilibrated lipid bilayer comprised of 100 POPC molecules. The system was solvated using the TIP3P water model and neutralized with sodium and chloride ions. Energy minimization and equilibration using NVT and NPT ensembles (1 ns) were carried out restraining the initial position of the molecule in the aqueous solution. The equilibrated systems were further used to pull the compound into the center of the POPC lipid bilayer during 500 ps with a pulling rate of 0.01 nm ps^{-1} and a harmonic force constant of 500 kJ mol^{-1} nm^{-2}. The pulling simulation was performed at 310 K and 1.0 bar using the Nosé-Hoover thermostat [10] and the semi-isotropic Parrinello–Rahman barostat.[11] Forty-three to forty-six configurations were selected along the z-axis reaction coordinate (ξ) for the umbrella sampling simulations. Each configuration was energy minimized and submitted to a restrained NPT equilibration before 10 ns of NPT production run, applying a harmonic force constant of 1000 kJ mol^{-1} nm^{-2} to the compound. The position and force data of all the configurations were evaluated with the Weighted Histogram Analysis Method (WHAM) [12] to generate the potential of mean force (PMF) profile. The structure with the lowest energy in the PMF profile of each compound was submitted to 300 ns non-restrained NPT production runs at 310 K and 1.0 bar.

<u>*Isolation of ventricular cardiomyocytes*</u>. Animal studies were conducted with approval from the University of Michigan Institutional Animal Care and Use Committee protocol PRO00010664. Cardiac myocytes were isolated from mice hearts using a modified Langendorff technique. Briefly, mice were intraperitoneal injected with 0.1 mL heparin (1000 IU/mL) 20 min before heart excision. Animals were anesthetized with 2% isoflurane and the heart was removed quickly from the chest and retrogradely perfused through the aorta at a constant flow (4 mL/min) at 37 ^{0}C for 5 min with a Ca^{2+}-free buffer containing 113 mM NaCl, 4.7 mM KCl, 1.2 mM $MgSO_4$, 0.6 mM Na_2HPO_4, 0.6 mM KH_2PO4, 10 mM $KHCO_3$, 12 mM $NaHCO_3$, 10 mM HEPES, 10 mM 2,3-butanedione monoxime (BDM, sigma) 30 mM taurine, and 5.5 mM glucose. All solutions were filtered (0.2 mm filter) and equilibrated with O_2 (100%) for at least 20 min before use. Enzymatic digestion was initiated by adding collagenase type II (773.4 U/mL; Worthington), trypsin (0.14 mg/mL), and $CaCl_2$ (12.5 mM) to the perfusion solution. After 5-6 min of digestion, the tissue was removed and placed in a 15*60 mm cell culture dish with stop solution (adding to the perfusion solution 10% fetal bovine serum and 12.5 µM $CaCl_2$), and gently dissociated with forceps. The isolated cardiac myocytes were collected, and calcium reintroduction was performed to a final concentration of 1 mM.

Preparation of cardiac myocytes for Ca^{2+} imaging studies. After myocyte dissociation and calcium reintroduction, the myocytes were allowed to equilibrate at room temperature for 10 min and transferred to a plating media solution with the following composition: culture media (see below) supplemented with 5% Fetal bovine serum (Sigma, F2442) and 25 µM S-(-)Blebbistatin (Sigma, B0560). Myocytes were counted using a hemocytometer, and approximately 20,000 cells were plated on previously 22×22 mm glass coverslips coated with laminin (40 µg/mL) using 6-well plates. The cells were allowed to attach for 40 min at room temperature. After cell attachment, the plating media was replaced with culture media with the following composition: 150 mL MEM +Hank's salts (Gibco, 500 mL 11575-032), 0.1% ITS liquid media supplement (100x, Sigma, I3146), 10 U/mL Penicillin/ 10 µg/mL Streptomycin (Invitrogen, 15070-063), 25 mM NaHCO3 (Sigma, S8875), 15 mM HEPES (Sigma H7006), 5 mM butanedione monoxime (BDM, sigma), and the cells were allowed to equilibrate for 20 min at room temperature.

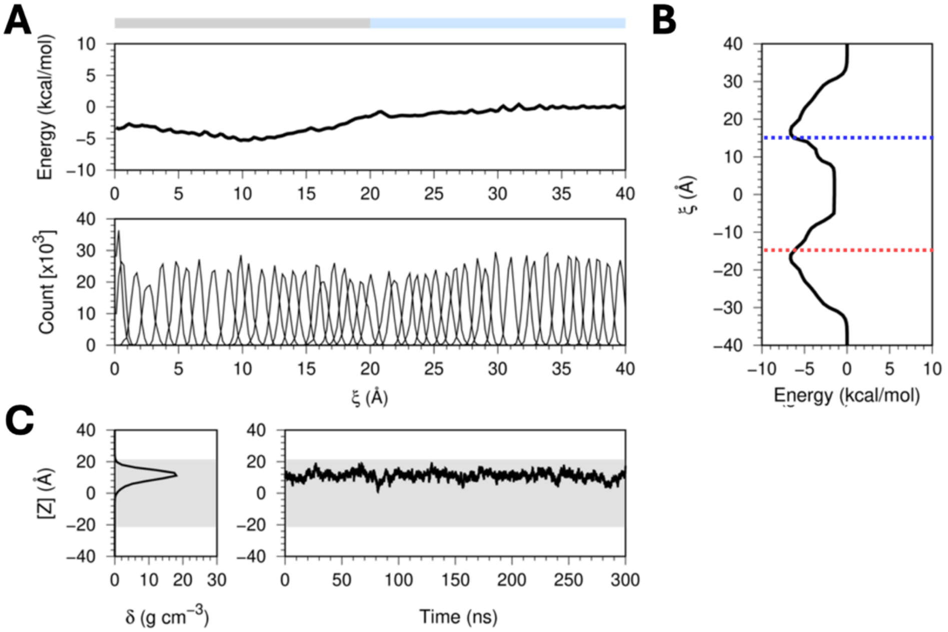

Fig. S1. Bilayer-crossing and ligand-membrane interactions of Yakuchinone A. (A) The potential of the mean force (PMF, top) and histogram (bottom) of Yakuchinone A along the normal z-axis of a POPC membrane based on umbrella sampling calculations; the distance along the z-axis was used here as a reaction coordinate (ξ). (B) Complementary bilayer-crossing free energy profile calculated with the PerMM server.[3] (C) Single-molecule atomistic simulations of ligand–membrane interactions of Yakuchinone A. The plots represent the position of a single molecule bound to a solvated POPC membrane. We show the relative density (δ) profile and time-dependent position of the molecule along the z-axis of the lipid–water system. The shaded area represents the location of the lipid bilayer.

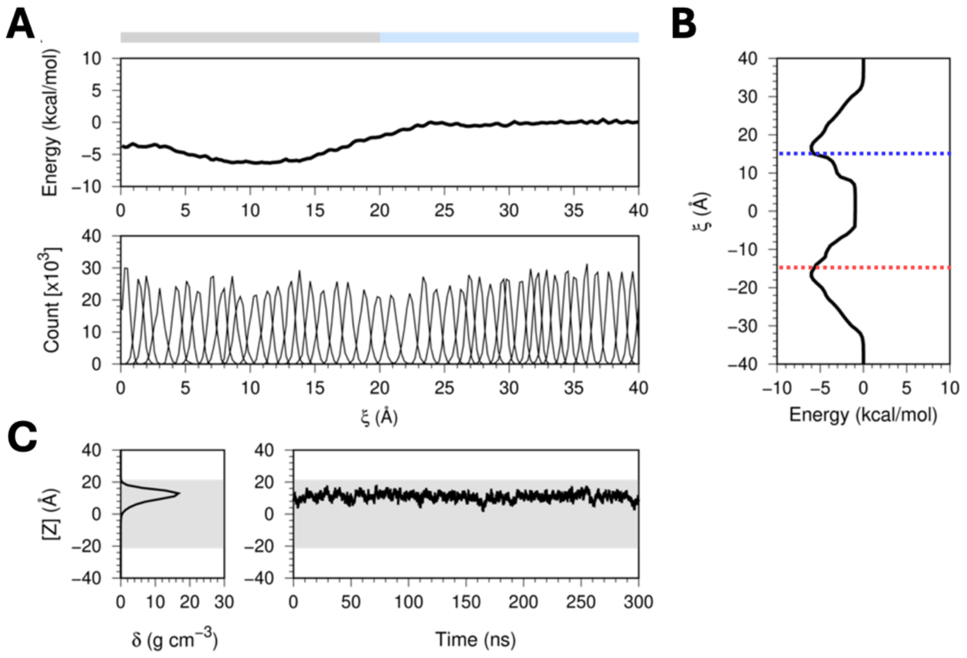

Fig. S2. Bilayer-crossing and ligand-membrane interactions of 6-paradol. (A) The potential of the mean force (PMF, top) and histogram (bottom) of 6-paradol along the normal z-axis of a POPC membrane based on umbrella sampling calculations; the distance along the z-axis was used here as a reaction coordinate (ξ). (B) Complementary bilayer-crossing free energy profile calculated with the PerMM server.[3] (C) Single-molecule atomistic simulations of ligand–membrane interactions of 6-paradol. The plots represent the position of a single molecule bound to a solvated POPC membrane. We show the relative density (δ) profile and time-dependent position of the molecule along the z-axis of the lipid–water system. The shaded area represents the location of the lipid bilayer.

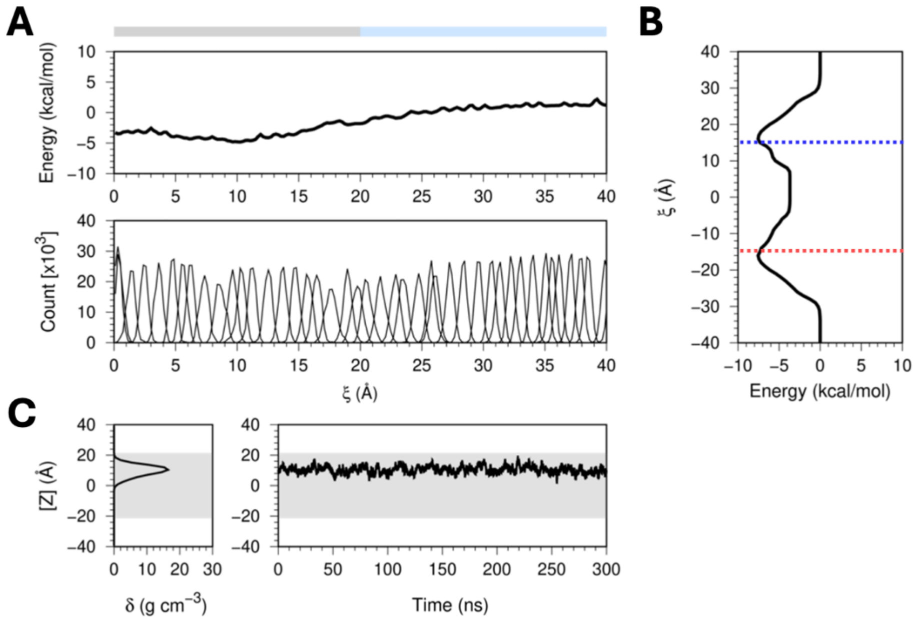

Fig. S3. Bilayer-crossing and ligand-membrane interactions of Alpinoid D. (A) The potential of the mean force (PMF, top) and histogram (bottom) of Alpinoid D along the normal z-axis of a POPC membrane based on umbrella sampling calculations; the distance along the z-axis was used here as a reaction coordinate (ξ). (B) Complementary bilayer-crossing free energy profile calculated with the PerMM server.[3] (C) Single-molecule atomistic simulations of ligand–membrane interactions of Alpinoid D. The plots represent the position of a single molecule bound to a solvated POPC membrane. We show the relative density (δ) profile and time-dependent position of the molecule along the z-axis of the lipid–water system. The shaded area represents the location of the lipid bilayer.

Supplementary references

[1] S. Kim, P. A. Thiessen, E. E. Bolton, J. Chen, G. Fu, A. Gindulyte, L. Han, J. He, S. He, B. A. Shoemaker, J. Wang, B. Yu, J. Zhang, S. H. Bryant, *Nucleic Acids Res* **2016**, *44*(D1), D1202-1213.

[2] N. M. O'Boyle, M. Banck, C. A. James, C. Morley, T. Vandermeersch, G. R. Hutchison, *J Cheminform* **2011**, *3*, 33.

[3] A. L. Lomize, J. M. Hage, K. Schnitzer, K. Golobokov, M. B. LaFaive, A. C. Forsyth, I. D. Pogozheva, *J Chem Inf Model* **2019**, *59*(7), 3094-3099.

[4] W. Fiehn, J. B. Peter, J. F. Mead, M. Gan-Elepano, *J Biol Chem* **1971**, *246*(18), 5617-5620.

[5] R. J. Bick, L. M. Buja, W. B. Van Winkle, G. E. Taffet, *J Membr Biol* **1998**, *164*(2), 169-175.

[6] K. Lindorff-Larsen, S. Piana, K. Palmo, P. Maragakis, J. L. Klepeis, R. O. Dror, D. E. Shaw, *Proteins* **2010**, *78*(8), 1950-1958.

[7] M. J. Abraham, T. Murtola, R. Schulz, S. Páall, J. C. Smith, B. Hess, E. Lindah, *SoftwareX* **2015**, *1-2*, 19-25.

[8] A. W. Sousa da Silva, W. F. Vranken, *BMC Res Notes* **2012**, *5*, 367.

[9] J. P. Jambeck, A. P. Lyubartsev, *J Chem Theory Comput* **2012**, *8*(8), 2938-2948.

[10] C. Braga, K. P. Travis, *J Chem Phys* **2005**, *123*(13), 134101.

[11] M. Parrinello, A. Rahman, *J Appl Phys* **1981**, *52*, 7182-7190.

[12] S. Kumar, J. M. Rosenberg, D. Bouzida, R. H. Swendsen, P. A. Kollman, *J Comput Chem* **1992**, *13*, 1011-1021.
